# Co-isolation of *Achromobacter* species and *Vibrio parahaemolyticus* from a case of chronic suppurative otitis media in a sea swimmer: a case report

**DOI:** 10.1186/s12879-025-12329-9

**Published:** 2025-12-27

**Authors:** Engy Aboelsaad, Hisham El-Badan, Eman A. Omran, Amira Amine, Laila El Attar

**Affiliations:** 1 Department of Microbiology, High Institute of Public Health, Alexandria University, El-Horreya Road 165, Alexandria, Egypt; 2 Department of Otorhinolaryngology, Faculty of Medicine, Alexandria University, Chamblion Street, Alexandria, Egypt

**Keywords:** Chronic suppurative otitis media, *Achromobacter*, *Vibrio parahaemolyticus*, Multi-drug resistance, Sea swimmers

## Abstract

**Background:**

*Achromobacter* species and *Vibrio parahaemolyticus* are environmental gram-negative bacteria that are rarely implicated in chronic suppurative otitis media (CSOM). *Achromobacter* species are emerging opportunistic pathogens, primarily associated with respiratory infections in immunocompromised individuals, while *Vibrio parahaemolyticus* is typically associated with seafood-related gastroenteritis. This case highlights the co-isolation of *Achromobacter* species and *Vibrio parahaemolyticus* from a patient with CSOM who frequently engaged in sea swimming, suggesting their potential role in CSOM pathogenesis and a possible environmental source of infection.

**Case presentation:**

A 15-year-old male, a sea swimmer, presented with persistent otorrhea and hearing impairment despite multiple courses of antibiotics. Otoscopic examination revealed tympanic membrane perforation with purulent discharge. Microbiological culture of the ear discharge isolated *Achromobacter* species and *Vibrio parahaemolyticus*, confirmed by matrix-assisted laser desorption/ionization time of flight-mass spectrometry (MALDI-TOF MS). *Achromobacter* species exhibited multidrug resistance, with susceptibility only to carbapenems, whereas *Vibrio parahaemolyticus* was susceptible to fluoroquinolones and tetracyclines. Targeted antimicrobial therapy led to clinical improvement.

**Conclusions:**

This case underscores the need to consider *Achromobacter* species and *Vibrio parahaemolyticus* as potential pathogens in non-resolving CSOM cases, especially in individuals exposed to marine environments. Their multidrug-resistance patterns highlight the importance of culture-based diagnosis and susceptibility testing for effective management. Further studies are needed to explore the epidemiology and clinical significance of *Achromobacter* species and *Vibrio parahaemolyticus* in ear infections.

## Background

Chronic suppurative otitis media (CSOM) is defined as a chronic inflammation of the mucoperiosteal lining of the middle ear cleft, characterized by recurrent or persistent ear discharge through a perforation of tympanic membrane (TM) [1]. It has been estimated that there are 31 million new cases of CSOM per year worldwide, with 22.6% of cases occurring in children less than 5 years old [2]. The disease is more prevalent in low socioeconomic groups because of malnutrition, overcrowding, poor hygiene, inadequate health care, and recurrent upper respiratory tract infections [3, 4].

The treatment of CSOM includes surgical as well medical measures. Topical quinolones are the treatment of choice [5, 6]. The increasing antibiotic resistance among bacterial isolates poses a therapeutic challenge to physicians. This is particularly important when antibiotics are prescribed empirically without antibiotic susceptibility testing. The periodic update of the spectrum of pathogens as well as their antibiotic susceptibility would be helpful in therapy and management of CSOM patients [3].

In CSOM, the causative bacterial agents may be aerobic (e.g. *Pseudomonas aeruginosa*,* Escherichia coli*,* Proteus mirabilis*,* Klebsiella* species (spp.), *Staphylococcus aureus*,* Streptococcus pyogenes*) or anaerobic (e.g. *Bacteroides* spp., *Peptostreptococcus*, *Cutibacterium*). Presence of anaerobes might lead to failure in the treatment of polymicobial CSOM [3, 7, 8]. Fungi have also been identified in cultures from patients with CSOM. The most commonly isolated fungi in CSOM are *Candida* and *Aspergillus* spp. The presence of fungi can be due to the treatment with antibiotic ear drops, which causes emergence of fungal microbiota [3].

*Achromobacter* is a genus of non-fermenting, gram-negative bacteria belonging to the order Burkholderiales. *Achromobacter xylosoxidans* “*A. xylosoxidans*” is the most often isolated *Achromobacter* spp. This bacterium is widely found in aquatic environments and has been associated with hospital outbreaks linked to contaminated fluids [9]. While it is most commonly found in the respiratory tracts of individuals with cystic fibrosis, *A. xylosoxidans* can also cause a variety of infections in individuals with other underlying health conditions [10].

*Achromobacter* spp. are infrequently associated with CSOM. These bacteria are often misidentified as other common non-fermenting gram-negative bacilli, such as *Pseudomonas aeruginosa*, *Stenotrophomonas maltophilia*, *Burkholderia cepacia complex*, and *Acinetobacter* spp., due to similarities in biochemical properties, and thus requires further diagnosis my other means such as matrix-assisted laser desorption/ionization time of flight-mass spectrometry (MALDI-TOF MS) or polymerase chain reaction (PCR). Their infrequent occurrence and constantly evolving taxonomy make it difficult to define their clinical features, acquisition risk factors, potential adverse outcomes, and the most effective treatments [11].

*Vibrio parahaemolyticus* “*V. parahaemolyticus*” is a gram-negative, halophilic bacterium commonly found in marine and coastal environments. It is the primary cause of acute gastroenteritis in humans, typically resulting from the consumption of raw, undercooked, or improperly handled seafood. In rare instances, *V. parahaemolyticus* can cause wound infections, ear infections, or septicemia in individuals with underlying medical conditions [12]. In this report, we describe a rare case of co-isolation of *Achromobacter* spp. and *V. parahaemolyticus* in CSOM.

## Case presentation

A 15-year-old male patient, with a history of regular swimming in the sea for the past 8 months, was admitted to the outpatient clinics of the Department of Otorhinolaryngology at Alexandria Main University Hospital. He presented with persistent left-sided otorrhea and hearing impairment despite multiple courses of topical empirical antibiotics (ciprofloxacin). The patient reported negative history for other acute/chronic diseases.

Otoscopic examination revealed tympanic membrane perforation with purulent discharge. Ear discharge was aseptically collected from the patient by the attending physician. The external ear was cleaned with 70% alcohol and three separate sterile swabs were used to collect the ear discharge [13]. The 1st swab was dipped immediately into a screw-capped sterile tube of Amies transport medium. The 2nd swab was inoculated immediately in Robertson’s cooked meat (RCM) broth and the 3rd swab was placed in a sterile tube [3]. The swabs were labeled and transported within one hour to the Microbiology Laboratory at the High Institute of Public Health.

The 1st swab was used for aerobic culture and was plated directly on each of 5% blood agar, MacConkey agar and chocolate agar plates. The blood and MacConkey agar plates were incubated aerobically while the chocolate plate was incubated in 5% CO_2_ atmosphere at 37 °C for 24–48 h [3]. The 2nd swab was used for anaerobic culture and was inoculated in Robertson’s cooked meat (RCM) and incubated at 37 °C for 72 h. On the 3rd day, subculture from RCM was made on neomycin blood agar with metronidazole 5 µg disc and incubated anaerobically at 37 °C for 72 h [14]. The 3rd swab was inoculated for fungal culture on two Sabouraud’s dextrose agar plates containing 0.05 mg/ml of chloramphenicol and then one of these plates was incubated at 28 °C and the other at 37 °C for one week [15].

Microbiological culture of the ear discharge on the blood, chocolate, and MacConkey agar plates resulted in the isolation of a mixed culture of *Achromobacter* spp. and *V. parahaemolyticus* confirmed by MALDI-TOF MS using the on-plate extraction method on the Bruker MALDI Biotyper system (version 3.1 database). *Achromobacter* spp. produced smooth, grayish-white, non-hemolytic colonies on blood agar and non-lactose fermenting colonies on MacConkey agar, while *V. parahaemolyticus* formed smooth, opaque, grayish colonies on blood agar and non-lactose fermenting colonies on MacConkey agar.

*Achromobacter* spp. and *V. parahaemolyticus* isolates were subjected to antimicrobial susceptibility testing using the VITEK 2 system with the AST-N335 card for gram-negative bacilli (bioMérieux) and results were interpreted according to CLSI guidelines [16]. The *Achromobacter* spp. isolate exhibited multidrug resistance, with susceptibility only to carbapenems, whereas *V. parahaemolyticus* was susceptible to fluoroquinolones and tetracyclines (Table 1). The initial empiric antimicrobial therapy with topical ciprofloxacin was changed. Based on the antimicrobial susceptibility testing, the patient received systemic therapy with a carbapenem to target *Achromobacter* spp. and an oral fluoroquinolone to cover *V. parahaemolyticus*. The regimen was continued for three weeks, during which clinical improvement was observed.

**Table 1:** Microbiological findings from ear swab cultures

Swab	Culture medium	Organism identified	Identification method	Antimicrobial susceptibility method	Key susceptibility results
Swab 1	Blood agar, MacConkey agar, and chocolate agar	- *Achromobacter* spp. - *V. parahaemolyticus*	MALDI-TOF MS	VITEK 2	- Susceptible to carbapenems - Susceptible to fluoroquinolones and tetracyclines
Swab 2	- Robertson’s cooked meat (RCM) - Neomycin blood agar with metronidazole 5 µg disc	-	-	-	-
Swab 3	Sabouraud’s dextrose agar	-	-	-	-

## Discussion and conclusions

This study reports a rare case of CSOM infection with *Achromobacter* spp. The same case yielded another uncommon pathogen, *V. parahaemolyticus.* This case highlights the importance of considering *Achromobacter* spp. and *V. parahaemolyticus* as potential pathogens in non-resolving CSOM cases, particularly in individuals who have been exposed to marine environments. The isolation of *V. parahaemolyticus* in this case is plausibly linked to the patient’s repeated exposure to seawater, consistent with its known ecology. In contrast, *Achromobacter* spp. may represent an opportunistic colonizer favored by prior broad-spectrum antibiotic courses. It is also possible that repeated antibiotic exposure suppressed more commonly encountered pathogens such as *Pseudomonas aeruginosa* or *Staphylococcus aureus*, which may have been underrepresented in culture results [9, 10].

*Achromobacter* spp. is an aerobic gram negative rod found in soil and water, including swimming pools, well water and chlorhexidine solutions [17]. In 2017, Cheong and Harding reported a case of septic arthritis of the temporomandibular joint as a rare complication of acute otitis media and was attributed to *A. xylosoxidans* [18]. Simialry, Ikari et al., reported a rare case of chronic mastoiditis and petrositis with viscous otorrhea caused by *A. xylosoxidans* [19]. In the present study, although MALDI-TOF MS identified the isolate as *A. xylosoxidans*, this method has known limitations in discriminating between closely related *Achromobacter* spp. Therefore, we refer to the isolate as *Achromobacter* spp. in this case report.

Treatment of *Achromobacter* spp. infections is often difficult and an optimal antimicrobial regimen has not been determined. Most *Achromobacter* spp. isolates have been found to be resistant to first- and second-generation cephalosporins, aminoglycosides, and narrow-spectrum penicillins; susceptible to sulfonamides, carbapenems, broad-spectrum penicillins, and third-generation cephalosporins; and variably susceptible to fluoroquinolones (such as ciprofloxacin) [20].

*V. parahaemolyticus* is a widespread bacterium found in temperate and tropical coastal regions globally. Infections are typically sporadic and linked to the consumption of raw or undercooked seafood, particularly in warmer months, though exposure to contaminated water can also lead to infections. Until the late 1960s, infections were mainly confined to Japan, but since 1969, *V. parahaemolyticus* has been reported in various locations, including the United States, Atlantic, Pacific, Gulf States, and Hawaii [21].

Various antibiotic resistance patterns have been observed in *V. parahaemolyticus* isolated from seafood and its environment across different countries. Recent studies found that *V. parahaemolyticus* strains in shrimp were resistant to colistin, ampicillin, and streptomycin but susceptible to other antibiotics. In the United States, strains from water and blue crab samples were resistant to several antibiotics, including cephalothin and cefoxitin [22].

The diagnosis of the mixed *Achromobacter* spp. and *V. parahaemolyticus* is difficult by conventional culture methods, and in the present study, an automated method (MALDI-TOF MS) was used to identify both organisms. Proper identification of such pathogens is important for such patient, since the infection would not respond to the commonly prescribed antibiotics in CSOM.

While both *Achromobacter* spp. and *V. parahaemolyticus* are typically capable of forming robust biofilms that contribute to their persistence and resistance, co-infection may potentially affect these processes. Within microbial communities, both intra- and inter-species interactions can occur, potentially affecting the progression of infections [23]. The interactions involving emerging pathogens like *Achromobacter* spp. have been seldom explored, likely due to the microorganism’s clinical relevance only becoming apparent in recent years. However, the increasing prevalence of *Achromobacter* spp. in cystic fibrosis patients, along with its frequent co-isolation with other pathogens, suggests that *Achromobacter* spp. are likely competing for space and nutrients [24]. Thus, co-infection with *Achromobacter* spp. and *V. parahaemolyticus* in CSOM may significantly alter their virulence and biofilm formation capabilities, which are critical factors in the pathogenicity of these bacteria. Therefore, further studies are needed to explore the molecular pathways underlying these interactions, which could inform new therapeutic strategies aimed at controlling infections caused by those mixed bacterial communities.

In conclusion, this case highlights the clinical relevance of detecting rare environmental pathogens in CSOM. The identification of *Achromobacter* spp. and *V. parahaemolyticus* underscores the importance of considering marine exposure as a potential risk factor in patients with CSOM. Furthermore, the presence of such atypical pathogens may have implications for empiric antimicrobial therapy, particularly in cases with a history of prolonged antibiotic use or environmental exposure. Awareness of these unusual etiologies can aid clinicians in guiding targeted diagnostic approaches and optimizing patient management. 

## Data Availability

The datasets analyzed during the current study are available from the corresponding author upon reasonable request.

## Abbreviations

*A. xylosoxidans*: *Achromobacter xylosoxidans*
AMUH: Alexandria Main University Hospital
CSOM: Chronic suppurative otitis media
HIPH: High Institute of Public Health
MALDI-TOF MS: Matrix-associated laser desorption ionization–time of flight mass spectrometry
RCM: Robertson’s cooked meat
Spp.: Species
TM: Tympanic membrane
*V. parahaemolyticus*: *Vibrio parahaemolyticus*

## References

[1] Abdullah M. Endoscopic assessment of the tympanic isthmus and tensor tympani fold as ventilation routes in cases of chronic suppurative otitis media. Faculty of Medicine, Alexandria University; 2018.

[2] Monasta L, Ronfani L, Marchetti F, Montico M, Brumatti L, Bavcar A, et al. Burden of disease caused by otitis media: systematic review and global estimates. PLoS ONE. 2012;7. 10.1371/journal.pone.0036226 PMC3340347 22558393

[3] Prakash R, Juyal D, Negi V, Pal S, Adekhandi S, Sharma M, et al. Microbiology of chronic suppurative otitis media in a tertiary care setup of Uttarakhand State, India. N Am J Med Sci. 2013;5(4):282–7. 23724403 10.4103/1947-2714.110436 PMC3662095

[4] Abdelshafy IA, Haleem AA, Khalil YA, Ghazal AA, Gaballah A, Saied A. Microbiology of chronic suppurative otitis Media,Study of the role of bacterial biofilm and fungal infection. J Otolaryngol Res. 2015;3(1):1–6.

[5] Harris AS, Elhassan HA, Flook EP. Why are ototopical aminoglycosides still first-line therapy for chronic suppurative otitis media? A systematic review and discussion of aminoglycosides versus quinolones. J Laryngol Otol. 2016;130(1):2–7. 26584651 10.1017/S0022215115002509

[6] Wang A, et al. Evaluation of surgical intervention for chronic suppurative otitis media in patients with hematologic diseases. Acta Otolaryngol. 2025;145(5):390–4. 40164142 10.1080/00016489.2025.2471940

[7] World Health Organization. Chronic suppurative otitis media: burden of illness and management options. Geneva: World Health Organization; 2004.

[8] Elmanama AA, Tayyem NEA, Allah SAN. The bacterial etiology of otitis media and their antibiogram among children in Gaza Strip, Palestine. Egypt J Ear Nose Throat Allied Sci. 2014;15(2):87–91.

[9] Kotani T, Inazaki T, Kasai H, Rakuman S, Suzuki K, Urushibara T. Pneumonia due to achromobacter xylosoxidans with a chronic course resembling non-tuberculous mycobacterial infection. Respirol Case Rep. 2024;12(2):e01287. 38314101 10.1002/rcr2.1287 PMC10831394

[10] Isler B, Kidd TJ, Stewart AG, Harris P, Paterson DL. Achromobacter infections and treatment options. Antimicrob Agents Chemother. 2020;64(11):e01025–20. 32816734 10.1128/AAC.01025-20 PMC7577122

[11] Bar-On O, Mei-Zahav M, Levine H, Mussaffi H, Blau H, Ben Zvi H, et al. The association of *Achromobacter xylosoxidans* airway infection with disease severity in cystic fibrosis. J Clin Med. 2025;14(7):2437. 40217889 10.3390/jcm14072437 PMC11989260

[12] Ceccarelli D, Hasan NA, Huq A, Colwell RR. Distribution and dynamics of epidemic and pandemic vibrio parahaemolyticus virulence factors. Front Cell Infect Microbiol. 2013;3:97. 24377090 10.3389/fcimb.2013.00097 PMC3858888

[13] Afolabi OA, Salaudeen AG, Ologe FE, Nwabuisi C, Nwawolo CC. Pattern of bacterial isolates in the middle ear discharge of patients with chronic suppurative otitis media in a tertiary hospital in North central Nigeria. Afr Health Sci. 2012;12(3):362–8. 23382753 10.4314/ahs.v12i3.18 PMC3557674

[14] Forbes B, Sahm D, Weissfeld A. Bailey and scott’s diagnostic microbiology. 14th ed. St. Louis: Mosby Inc; 2017.

[15] Rippon J. Medical mycology. 2nd ed. Canada: Saunders company; 1982. pp. 772–95.

[16] Clinical and Laboratory Standards Institute (CLSI). Performance standards for antimicrobial susceptibility testing. 34th ed. Suppl M100 CLSI; 2024.

[17] Yabuuchi E, Oyama A. Achromobacter xylosoxidans n. sp. from human ear discharge. Jpn J Microbiol. 1971;15(5):477–81. 5316576 10.1111/j.1348-0421.1971.tb00607.x

[18] Cheong RC, Harding L. Septic arthritis of the temporomandibular joint secondary to acute otitis media in an adult: a rare case with *Achromobacter xylosoxidans*. Case rep Otolaryngol. 2017;2017. 10.1155/2017/3641642 PMC5387829 28458938

[19] Ikari E, Komune N, Nakagawa T. Chronic mastoiditis and petrositis with viscous otorrhea caused by achromobacter xylosoxidans: case report and literature review. Otolaryngol Case Rep. 2018;7:3–6.

[20] Rolston KV, Messer M. The in-vitro susceptibility of alcatigenes deni-trificans subsp. Xylosoxidans to40 anti-microbial agents. J Antimicrob Chemother. 1990;26(6):857–9. 2081727 10.1093/jac/26.6.857

[21] Baker-Austin C, Oliver JD, Alam M, Ali A, Waldor MK, Qadri F, Martinez-Urtaza J. Vibrio spp. Infections. Nat Reviews Disease Primers. 2018;4(1):1–9. 10.1038/s41572-018-0005-8 30002421

[22] Ngasotter S, Mukherjee S, Singh SK, Bharti D, Haque R, Varshney S, Nanda C, Waikhom D, Devi MS, Singh AS. Prevalence, Virulence, and antibiotic resistance of vibrio parahaemolyticus from seafood and its environment: an updated review. Mediterr J Infect Microb Antimicrob. 2022;11(1):1–1.

[23] Magalhães AP, Lopes SP, Pereira MO. Insights into cystic fibrosis polymicrobial consortia: the role of species interactions in biofilm development, phenotype, and response to in-use antibiotics. Front Microbiol. 2017;7:2146. 28133457 10.3389/fmicb.2016.02146 PMC5233685

[24] Sandri A, Haagensen JAJ, Veschetti L, Johansen HK, Molin S, Malerba G, Signoretto C, Boaretti M, Lleo MM. Adaptive interactions of *Achromobacter* spp. With *Pseudomonas aeruginosa* in cystic fibrosis chronic lung Co-Infection. Pathogens. 2021;10(8):978. 34451442 10.3390/pathogens10080978 PMC8400197

